# Hospital Meetings

**Published:** 1894-12-01

**Authors:** 


### HOSPITAL FESTIVALS AND MEETINGS.

### CHELSEA HOSPITAL FOR WOMEN.

On Wednesday, November 28th, a special meeting of Governors was held at the Chelsea Hospital for Women.

Sir Algernon Borthwick was asked to take the chair, and proceeded to recall to those present the various circumstances which had led to the present awkward position. At the last special meeting of the Governors the old Board of Management had resigned in a body, and had been re-elected, the number of votes being fourteen to eight. Owing to further "misunderstandings," the Board now again placed their resignations in the hands of the Governors, as also had the medical staff, and the present meeting was called in order to elect a new Board, and to receive certain suggestions from the late medical staff. He was prepared for his part to offer himself for re-election, feeling that the interest of the poor women involved demanded that everyone should do all in their power for the hospital. A list of proposed names for the new Board had been prepared, and consisted of the following: Sir Algernon Borthwick, Bart., M.P., Mr. W. L. A. Burdett-Coutts, M.P., Mr. Thomas W. Brookes, Mr. A. J. Wright-Biddulph, J.P., D.L., Mr. James Bailey, Mr. Robert J. Beadon, Colonel Colville, Mr. T. Dyer Edwards, J.P., Mr. Charles Fish, Mr. George Hilditch, Mr. J. L. Langman, Mr. Samuel Lithgow, Sir Oswald Moseley, Mr. J. Cumming Macdona, M.P., Mr. Ernest Oppenheim, Major Probyn, L.C.C., Mr. Holmes Stone, Colonel Shipway, Mr. J. G. Woodroffe, and Mr. J. S. Wood.

After a proposal by Dr. Travers that the names should be voted for singly, which was negatived decidedly by the meeting, votes were taken for the new members *en bloc*; and the new Board of Management now stands elected as above by 71 votes to 9.

Some discussion arose on the vexed point of the non-election of certain members of the late medical staff, on the ground of their being "general practitioners," Lord Cadogan, as chairman of the late Board, saying that this was the only condition made, and that the secretary had been desired to mention it in the advertisement inviting applicants to apply for the vacant posts, but that he had apparently omitted to do so. This did not appear in the minutes, however, and no clear explanation seemed to be forthcoming.

Dr. Duncan, on behalf of the medical staff who have resigned in a body in order to leave the hands of the Board free in the election of a fresh staff, but who will carry on the work of the hospital until that time, suggested that in advertising for gentlemen to come forward for election the advertisement should not only state what were the necessary qualifications, but should also mention the disqualifications, and also suggested for the consideration of the Board that it would be well to obtain the assistance in these elections of some leading London obstetric physicians and surgeons.

In conclusion Lord Cadogan was asked to reconsider his resignation from the Board, and after stating as his reason for resigning that he felt he must stand shoulder to shoulder with his colleagues, he asked that his final decision might be postponed for a few days.

The meeting concluded with a hearty vote of thanks to Sir Algernon Borthwick.

